# Epley’s Influence on Horizontal Canal BPPV Variants

**DOI:** 10.3390/audiolres15020025

**Published:** 2025-03-07

**Authors:** Olivia Kalmanson, Carol Foster

**Affiliations:** Department of Otolaryngology, University of Colorado Anschutz School of Medicine, Aurora, CO 80045, USA; olivia.kalmanson@cuanschutz.edu

**Keywords:** benign paroxysmal positional vertigo, epley, cupulolithiasis, canalith jam

## Abstract

Dr. Epley has been instrumental in defining the mechanisms and treatment of BPPV variants, including those of the horizontal canals. Cupulolithiasis is a horizontal canal BPPV variant usually defined as direction-changing apogeotropic nystagmus. In recent years, the favored cupulolithiasis mechanism of otoconia adhering persistently to the cupula has been called into question. Epley was the first to propose mechanistic theories which better match the most recent evidence. From the beginning, he has demonstrated mastery over the semicircular canal pathology and otoconial mechanics.

## 1. Introduction

Dr. Epley’s unique contributions to our understanding of benign paroxysmal positional vertigo (BPPV) are numerous, of great consequence, and have revealed insights into the condition that were ahead of his time. Prior to 1979, the pathology of classic BPPV was not understood. After its initial description in 1921 [1], BPPV was usually ascribed to utricular disease. However, the utricular theory failed to explain the characteristic nystagmus of the disorder. Schuknecht was the first to postulate a semicircular canal-based mechanism for BPPV. In the early 1960s, he correctly theorized that utricular damage could release otoconia that could then affect the cupula of the posterior semicircular canal. These relatively heavy particles had the potential to render the cupula gravity-sensitive and better explained the characteristic torsional nystagmus, which could not be due to the utricle [2]. These ideas represented positive progress toward our current understanding of BPPV. When his definitive theory was published in 1969, however, he attributed the nystagmus to otoconia attached to the utricular side of the cupula or directly impacting it, which he called cupulolithiasis [3]. His strongest evidence was two temporal bone specimens showing deposits that appeared to adhere to cupulae that were retracted and detached from the ampullary wall. While Schuknecht later corrected his theory by placing particles on the canal side of the cupula, his original theory held sway for many years and was widely accepted. Cupulolithiasis, like the utricular theory, also had shortcomings and was fated to be disproven as the mechanism for classic BPPV. The greatest problem was that otoconia adhering to the cupula could not explain the short duration and fatiguability of the accompanying nystagmus. If particles strongly adhered to the cupula, the nystagmus would have been prolonged and non-fatiguing.

It was Hall, Ruby and McClure who, in 1979, published their seminal paper based on mechanical experiments by Joseph McClure that freely moving particles in the semicircular canal explained classic BPPV better than the cupulolithiasis theory [4]. Otoconia moving with gravity, called canalithiasis, explained all aspects of nystagmus in classic BPPV. This new and ultimately correct theory, however, was unable to displace Schuknecht’s cupulolithiasis theory as the cause of BPPV for several more years.

Epley had a deep understanding of inner ear anatomy and immediately saw the key implication of McClure’s canalithiasis theory. Free particles that move under the influence of gravity should be able to be rotated out of the semicircular canal. Within months, he had devised a treatment maneuver that was highly effective and ultimately revolutionized the treatment of BPPV [5,6]. Although his data were presented at meetings, they went unpublished for over a decade and so the cupulolithiasis explanation continued to attract adherents. In 1992, two key papers were published. Epley introduced his maneuver to the world [7], and Parnes and McClure presented operative proof of otoconia moving in the canal [8]. These papers cracked the cupulolithiasis edifice, and thereafter, posterior canal BPPV was increasingly explained by freely moving particles in the canal. Epley’s repositioning maneuver, although slow to achieve acceptance, had such a high success rate that it provided proof of McClure’s theory. Numerous studies thereafter supported canalithiasis, and it is now the accepted mechanism of most forms of fatigable BPPV [9,10,11].

Epley’s influence, while key to explaining and treating posterior canal BPPV, did not end there. He understood and published anatomic insights such as those into the orientation of cupulae within the ampullae, the vector of ocular rotation for each canal, and the effect of gaze orientation on the nystagmus of BPPV [5]. He noted major complications of maneuvers, such as multiple canal BPPV, reflux during maneuvers and canal switch [5]. He also described a new problem that he called canalith jam, with wedging of canaliths in the ampulla or at narrowings in the canals [5,12]. Unlike his revolutionary maneuver, however, this concept was only infrequently cited for many years.

In 1985, the geotropic variant of horizontal canal BPPV was described and correctly attributed to canalithiasis [13]. Cupulolithiasis was still occasionally invoked as an explanation for rare, non-fatiguing positional nystagmus. However, when the apogeotropic form of horizontal canal BPPV was identified, this more persistent form was attributed to cupulolithiasis, giving new life to the old theory [14]. A canalithiasis mechanism for apogeotropic horizontal canal BPPV had already been put forward by Nuti, Vannuchi and Pagnini—periampullary canalithiasis [15], but went largely unappreciated by otologists. In spite of this viable alternative theory, many articles to the present continue to treat apogeotropic horizontal canal BPPV as synonymous with cupulolithiasis.

Epley’s concept of canalith jam provides another explanation for apogeotropic horizontal canal BPPV that upholds canalithiasis as the cause of all forms of BPPV and obviates the need for cupulolithiasis as a separate mechanism [16]. In this review, we outline the contributions Epley has made in understanding the BPPV variants of cupular impingement and canalith jam.

## 2. Materials and Methods

A review was undertaken of the BPPV variant classically known as cupulolithiasis. We performed literature reviews in PubMed and Google Scholar using three separate keywords, “cupulolithiasis”, “apogeotropic [and] benign”, and “canalith jam”. A total of 202 unique full-text articles were available in English, which were read in full to obtain a complete history on the subject. An additional three references relevant to the subject and important for background reading, predominantly on the anatomy and physiology of the cupula and semicircular canals, were identified from the reference lists of the original 202 articles and included as well, totaling 205 different articles.

## 3. History of Cupulolithiasis and Canal Jam Mechanistic Theories

### 3.1. Epley, Cupular Impact Theory, and Periampullary Canalithiasis

After the freely moving particle mechanism was comfortably established for classic BPPV, other variants were brought to light along with new theories to explain the nystagmus. Persistent apogeotropic direction-changing nystagmus (horizontal canal BPPV) was recognized as the sole remaining example of cupulolithiasis [14], in spite of the fact that periampullary free particles equally explained the condition. Epley then provided a third mechanism. This was the concept of cupular impingement without adherence to the cupula [5]. Schuknecht had also mentioned this possibility as a mechanism for classic BPPV, but this was discarded once his cupulolithiasis theory was accepted. Epley described a half-Hallpike position resulting in otoconia resting on the cupula, which caused prolonged apogeotropic nystagmus. Upon rotating the head 180 degrees, the otoconia would roll away, causing the nystagmus to cease, which suggested nonadherent cupular impingement. This theory could explain apogeotropic nystagmus in one position, but not its reversal on rolling the head to the opposite side. Although Nuti et al. published proof that the majority of cases of apogeotropic nystagmus are explained by periampullary canalithiasis at that time, Epley and most other North American otologists were not aware of the paper [15]. It was not until several years later that a paper demonstrated freely moving otoconia near the ampulla of the horizontal SCC would cause apogeotropic nystagmus [17]. Since Epley’s intra-ampullary particles would become periampullary as soon as they exited the ampulla, Nuti et al.’s theory was then capable of explaining apogeotropic nystagmus without the need for any attachment of particles to the cupula. Further, such periampullary particles would be refractory to classic 180-degree maneuvers because they would fail to clear the full 270-degree length of the affected canal [15,17]. The combination of periampullary canalithiasis with or without intra-ampullary cupular impingement could then cause both major signs that were ascribed to cupulolithiasis of the horizontal canal: the apogeotropic persistent nystagmus and the poor response to maneuvers used at that time.

A recent critical review of cupulolithiasis suggests that the majority of cases published as cupulolithiasis (61%) are better explained by periampullary canalithiasis [16]. The implication is that papers studying cupulolithiasis are largely contaminated with cases of periampullary and intra-ampullary canalithiasis. Experimental animal models of cupular responses show that cupulolithiasis should result in a low-grade persistent nystagmus [18,19,20]. Many papers, including the first paper attributing apogeotropic nystagmus to cupulolithiasis [14], show a peaked pattern of much higher velocity nystagmus suggesting that particles are freely moving [16].

Now that periampullary forms of horizontal canal BPPV are better understood, newer maneuvers have been developed that more fully clear the horizontal canal, such as the inverted Gufoni maneuver followed by the Gufoni maneuver [21,22]. Yet, a new problem then arose: some patients failed to resolve even with newer maneuvers. While this was uncommon, it provided yet another justification for retaining cupulolithiasis as a mechanism: the theory that particles were so adherent to the cupula that they could not be dislodged. Epley, however, had already published another mechanism that had the potential to explain these resistant forms of apogeotropic direction changing nystagmus in horizontal canal BPPV [6].

### 3.2. Epley and Canal Jam

Dr. Epley was the first to suggest canal jam as another mechanism to explain clinical findings in BPPV [5,6]. He noted sudden distress with a direction-fixed nystagmus while performing maneuvers. He theorized that during maneuvers, the mass of moving particles could become wedged at the junction of both vertical canals with the common crus. The symptoms were similar to canal plugging. The findings he reported for canal jam include prolonged direction-fixed nystagmus with canal paresis and impaired vestibular head-impulse test responses. Others ultimately followed suit and identified cases of canal jam in their practices [23]. Epley recommended reversing the maneuver to resolve the jam. This was seen as a posterior canal disorder, and did not apply directly to the horizontal canal, which lacks a common crus.

Epley also understood, however, that all canals have areas of narrowing that could entrap large masses of particles [24]: the ampullary-canal junction. If particles can freely move in and out of the ampulla, it becomes possible for masses of particles to block this junction like a check valve [25]. In the horizontal canal, a large mass could then move back and forth between the cupula and the ampullary canal junction as the head is turned horizontally. It is also understood that otoconia do not necessarily move as independent masses like beads, but can exist as sheets of otoconial membrane embedded with otoconia [26]. These large and irregular masses provide a mechanism for blockages at narrowings in the canal that can then be resistant to maneuvers.

In recent years, several cases of mobile or reversible canal jams have been published [25,27,28,29,30], suggesting a blur between two of Epley’s own theories: cupular impingement and canal jam. If masses of otoconial debris accumulate in the ampulla, they can adhere to each other and grow larger than the canal diameter at its narrowest point. Such an entrapped mass would then oscillate between the cupula and the narrowing in the canal, unable to exit with head turns. The nystagmus resulting from this oscillation should result in apogeotropic nystagmus, as noted in a case report [25]. The otoconial mass at one extreme impinges directly on the cupula, as Epley envisioned, and as the head is turned to the opposite side, the cupula deviates toward the moving mass, reversing the nystagmus. As the otoconia move into the canal and then wedge, this results in a relative vacuum holding the cupula in its deviated position and causing the nystagmus to persist [16]. The mounting evidence supporting mobile or reversible canal jams as an explanation for persistent apogeotropic nystagmus further lessens the need for cupulolithiasis as a mechanism. This suggests that all BPPV variants may lie on a spectrum of the freely moving particle theory, of which Epley formed the foundation [16].

## 4. Discussion

While numerous authors over time continued to cite adherent cupulolithiasis as the etiology for horizontal canal BPPV variants in an effort to explain the resultant nystagmus, all clinical findings can be explained by a spectrum of freely moving particle pathology. This spectrum includes everything between the mobile individual particles of classic BPPV through a fixed canal jam when a large particle burden is present and blocks the canal [16,31]. Epley’s theory of nonadherent cupular impingement paired with his description of canal jam have been combined into a theory of reversible canal jam that does not require adherence of otoconia to the cupula as a mechanism for apogeotropic nystagmus [16,25,27,28,29,30]. Rather than postulating two different mechanisms to explain BPPV, prolonged adherence to the cupula is no longer required since all signs of the disorder can be explained by mobile canaliths. The exact mechanisms of rare forms of BPPV could be proven when real-time micro-imaging modalities become available. There are also likely to be forms of direction-changing positional nystagmus that are not BPPV-related, and their mechanism awaits further study.

There are several major flaws with the theory behind cupulolithiasis, which are described in detail elsewhere [16]. An updated summary of the arguments outlined in that review follows, including relevant publications released in the interim.

The first flaw in the cupulolithiasis theory lies in the affinity for otoconia to adhere to the cupula so strongly that they could not be dislodged. Histologic images have repeatedly demonstrated that in all three canals, the ampullae open widely into the vestibule [24] and are likely constantly bathed in otoconia. If the cupulae were sticky, cupulolithiasis would be the most common form of BPPV in all canals. The historic histologic finding of otoconia adherent to the cupula, published by Schuknecht when he theorized cupulolithiasis to be the cause of classic BPPV, were in fact artifactual [3]. The cupula is a sheet completely spanning the cross-sectional area of the ampulla. Upon fixation and processing, the cupula releases from the wall and shrinks down near the crista ampullaris. Schuknecht unknowingly observed this in the form of dense deposits adhered to the ruptured or torn edge, a distinctly non-physiological state. Since both of the patients he reported had classic symptoms of posterior canal BPPV that we now know is due to mobile canaliths, it is difficult to explain how they could also have had cupulolithiasis of the posterior canal. Moreover, in order to manifest symptoms of classic BPPV, the cupula must have been intact premortem, because detached cupula are incapable of signaling motion [32]. We can thus be certain that the cupula detached from the ampullae were postmortem artifact. This landmark histologic image should therefore no longer be used as evidence for otoconial adherence to the cupula. There is also no physiologic rationale for the cupulae to have adherent qualities, since it is designed to function solely as an acceleration sensor and is disabled if it were to function as a gravity sensor. Cupulin is the primary glycoprotein composing the cupular membrane. It is known for its elastic properties thereby giving the cupula a self-cleaning effect because it can flex and release potentially adherent substances [33,34]. As such, while the otoconia may slough off from the utricle with attached pieces of otoconial membrane, which is known to be highly adherent, otoconial masses would not have a special predilection for the cupula. They would more likely adhere—based on total surface area—to the extensive walls of the membranous labyrinth or to each other rather than to the much smaller and elastic, constantly flexing cupula. Adherence of otoconia and detached otoconial membranes to the canal walls or to each other are more aligned with the canal jam mechanisms, and they are supported by micro-computed tomography data described below [29,35].

Second, as described in Section 3.2 above, cupulolithiasis has been over-cited as the cause of direction-changing apogeotropic nystagmus. Nuti et al. was the first to publish that fatigable apogeotropic nystagmus can be explained by periampullary free-floating canalithiasis [15]. The prevalence of cupulolithiasis in the literature likely lies in the confusion over the diagnosis, which should require a persistent or non-fatigable quality in addition to mere apogeotropic nystagmus. Many recent articles used apogeotropic nystagmus as the only criteria for a cupulolithiasis diagnosis without citing periampullary canalithiasis as a possibility. A recent review noted that one-third of papers on apogeotropic nystagmus failed to mention periampullary canalithiasis in the differential diagnosis [16]. This has resulted in a profusion of papers seeking to differentiate particles adherent to the utricular side of the cupula from those on the canal side, and to provide treatment maneuvers for each. This is unnecessary if the issue is periampullary canalithiasis.

Evidence for periampullary (apogeotropic) canalithiasis lies in its conversion to geotropic canalithiasis after maneuvers [36,37,38,39]. Periampullary (apogeotropic) canalithiasis cannot be resolved solely with maneuvers designed for the geotropic form of horizontal canal BPPV because the particles are further from the vestibule in the periampullary form [17,22,40,41,42]. The appearance that a case is refractory to maneuvers results when the proper sequence is not used to completely clear the canal, and this is easily mistaken for adherence to the cupula. Newer maneuvers, including the inverted Gufoni and Vannuchi Asprella maneuvers can resolve these forms, as can classic Lempert barbecue rolls [21,22]. To broaden the discussion of the differential diagnosis of direction-changing apogeotropic nystagmus, central pathologies should also be included. Multiple examples have been published of strokes mimicking horizontal canal cupulolithiasis [43,44]. Central pathologies must also be considered and ruled out during the diagnostic workup of refractory BPPV variants.

Third, imaging studies, while somewhat limited, do not support the theory of cupulolithiasis. Micro-computerized tomography studies have demonstrated otoconial accumulation on the semicircular canal walls—not on the cupula. They also report narrowed canal lumens and frequent canal jam in patients diagnosed with cupulolithiasis. This suggests that many cases attributed to cupulolithiasis may be better explained by canal jam following canalithiasis. In contrast, there was less otoconial burden and fewer canal jams in patients diagnosed with BPPV due to uncomplicated canalithiasis and in controls without vertigo [29,35].

Fourth, reversible canal jam and cupulolithiasis cannot be differentiated based on the characteristics of the nystagmus and the response to maneuvers since both would theoretically cause an apogeotropic nystagmus that is refractory to maneuvers [25,30]. Even the presence of canal paresis, attributed by Epley to jam, cannot distinguish between the two. A bullfrog model of cupulolithiasis in which the investigators placed otoconia onto the cupula resulted in a decreased responsiveness to motion regardless of head position, attributed to increased cupular mass. However, this also occurred when jamming the particles into the canal, suggesting that features of canal jam can be seen in either disease [20]. Numerous new maneuvers that purport to dislodge particles adherent to either side of the cupula also involve head motions that are likely to help break up persistent canal jams.

The current literature and limitations in micro-computed tomography do not yet allow differentiation of the two pathophysiologic mechanisms. Only through evidence-based deductive reasoning and improvements in imaging will practitioners finally determine which mechanism is more logical or likely.

## 5. Conclusions

In summary, Epley’s contributions to the understanding of BPPV mechanisms through freely moving otoconia, including sufficient otoconia to create a jam, continue to elegantly explain all forms of horizontal canal BPPV without the need for adherent cupulolithiasis as a separate entity. Amid often chaotic trends in the literature during which time cupulolithiasis fell in and out of favor to explain different BPPV variants, Epley remained steadfast in his ability to thoughtfully consider vestibular physiology and draw evidence-based conclusions in both his clinical realm and research endeavors.

Epley demonstrated unmatched mastery over the mechanics of otoconia and the semicircular canals. His interpretation of nystagmus patterns have contributed immensely to the understanding of BPPV and its variants, including horizontal canal BPPV variants exhibiting apogeotropic nystagmus. His proof of McClure’s theory that freely moving otoconia dominate the mechanisms of BPPV and its variants has withstood the test of time.

## Data Availability

No new data were created or analyzed in this study. Data sharing is not applicable to this article.

## References

[1] Bárány R. (1921). Diagnose von Krankheitserscheinungen im Bereiche des Otolithenapparatus. Acta Otolaryngol..

[2] Baloh R.W. (2001). Harold Schuknecht and pathology of the ear. Otol. Neurotol..

[3] Schuknecht H.F. (1969). Cupulolithiasis. Arch. Otolaryngol..

[4] Hall S.F., Ruby R.R., McClure J.A. (1979). The mechanics of benign paroxysmal vertigo. J. Otolaryngol..

[5] Epley J.M. (1997). Caveats in particle repositioning for treatment of canalithiasis (BPPV). Oper. Tech. Otolaryngol. Head Neck Surg..

[6] Epley J.M. (2001). Human Experience with Canalith Repositioning Maneuvers. Ann. N. Y. Acad. Sci..

[7] Epley J.M. (1992). The canalith repositioning procedure: For treatment of benign paroxysmal positional vertigo. Otolaryngol. Head Neck Surg..

[8] Parnes L.S., Mcclure J.A. (1992). Free-Floating endolymph particles: A new operative finding during posterior semicircular canal occlusion. Laryngoscope.

[9] Moriarty B., Rutka J., Hawke M. (1992). The incidence and distribution of cupular deposits in the labyrinth. Laryngoscope.

[10] Welling D.B., Parnes L.S., O’Brien B., Bakaletz L.O., Brackmann D.E., Hinojosa R. (1997). Particulate matter in the posterior semicircular canal. Laryngoscope.

[11] Bachor E., Wright C.G., Karmody C.S. (2002). The incidence and distribution of cupular deposits in the pediatric vestibular labyrinth. Laryngoscope.

[12] Epley J.M. (1995). Canalith Repositioning. Otolaryngol. Head Neck Surg..

[13] McClure J.A. (1985). Horizontal canal BPV. J. Otolaryngol..

[14] Baloh R.W., Yue Q., Jacobson K.M., Honrubia V. (1995). Persistent direction-changing positional nystagmus: Another variant of be-nign positional nystagmus?. Neurology.

[15] Nuti D., Vannucchi P., Pagnini P. (1996). Benign paroxysmal positional vertigo of the horizontal canal: A form of canalolithiasis with variable clinical features. J. Vestib. Res..

[16] Kalmanson O., Foster C.A. (2023). Cupulolithiasis: A Critical Reappraisal. OTO Open.

[17] Asprella Libonati G. (2005). Diagnostic and treatment strategy of lateral semicircular canal canalolithiasis. Acta Otorhinolaryngol. Ital..

[18] Valli P., Botta L., Zucca G., Valli S., Buizza A. (2008). Simulation of cupulolithiasis and canalolithiasis by an animal model. J. Vestib. Res..

[19] Ichijo H. (2013). Cupulolithiasis of the posterior semicircular canal. Am. J. Otolaryngol..

[20] Inagaki T., Suzuki M., Otsuka K., Kitajima N., Furuya M., Ogawa Y., Takenouchi T. (2006). Model experiments of BPPV using isolated utricle and posterior semicircular canal. Auris Nasus Larynx.

[21] Asprella Libonati G., Gagliardi G., Cifarelli D., Larotonda G. (2003). “Step by step” treatment of lateral semicircular canal canalolithiasis under videonystagmoscopic examination. Acta Otorhinolaryngol. Ital..

[22] Lee H.J., Jeon E.-J., Nam S., Mun S.-K., Yoo S.-Y., Bu S.H., Choi J.W., Chung J.H., Hong S.M., Lee S.-H. (2023). Treatment Efficacy of Various Maneuvers for Lateral Canal Benign Paroxysmal Positional Vertigo With Apogeotropic Nystagmus: A Randomized Controlled Trial. Clin. Exp. Otorhinolaryngol..

[23] von Brevern M., Clarke A.H., Lempert T. (2001). Continuous vertigo and spontaneous nystagmus due to canalolithiasis of the horizontal canal. Neurology.

[24] Southern Illinois University Histology. https://histology.siu.edu/ssb/EE006b.htm.

[25] Kalmanson O.A., Aasen D.M., Gubbels S.P., Foster C.A. (2021). Reversible Canalith Jam of the Horizontal Semicircular Canal Mimicking Cupulolithiasis. Ann. Otol. Rhinol. Laryngol..

[26] Kao W.T.K., Parnes L.S., Chole R.A. (2016). Otoconia and otolithic membrane fragments within the posterior semicircular canal in benign paroxysmal positional vertigo. Laryngoscope.

[27] Comacchio F., Poletto E., Mion M. (2018). Spontaneous Canalith Jam and Apogeotropic Horizontal Canal Benign Paroxysmal Positional Vertigo: Considerations on a Particular Case Mimicking an Acute Vestibular Deficit. Otol. Neurotol..

[28] Schubert M.C., Helminski J., Zee D.S., Cristiano E., Giannone A., Tortoriello G., Marcelli V. (2020). Horizontal semicircular canal jam: Two new cases and possible mechanisms. Laryngoscope Investig. Otolaryngol..

[29] Yamane H., Konishi K., Anniko M. (2021). Visualization of horizontal canal benign paroxysmal positional vertigo using 3DCT imaging and its assessment. Acta Otolaryngol..

[30] Gufoni M., Casani A.P. (2022). The clinical significance of direction-fixed mono-positional apogeotropic horizontal nystagmus. Acta Otorhinolaryngol. Ital..

[31] Imai T., Takeda N., Ikezono T., Shigeno K., Asai M., Watanabe Y., Suzuki M. (2017). Classification, diagnostic criteria and management of benign paroxysmal positional vertigo. Auris Nasus Larynx..

[32] Rabbitt R.D., Breneman K.D., King C., Yamauchi A.M., Boyle R., Highstein S.M. (2009). Dynamic displacement of normal and detached semicircular canal cupula. J. Assoc. Res. Otolaryngol..

[33] Dernedde J., Weise C., Müller E.-C., Hagiwara A., Bachmann S., Suzuki M., Reutter W., Tauber R., Scherer H. (2014). Cupulin is a zona pellucida-like domain protein and major component of the cupula from the inner ear. PLoS ONE.

[34] Rabbitt R.D. (2019). Semicircular canal biomechanics in health and disease. J. Neurophysiol..

[35] Horii A., Kitahara T., Osaki Y., Imai T., Fukuda K., Sakagami M., Inohara H. (2010). Intractable benign paroxysmal positioning vertigo: Long term follow up and inner ear abnormality detected by three dimensional magnetic resonance imaging. Otol. Neurotol..

[36] Vats A.K. (2022). A case of apogeotropic horizontal canal benign paroxysmal positional vertigo that transformed to the geotropic variant during treatment with Appiani maneuver, followed by successful treatment with Gufoni maneuver. Physiother. Theory Pract..

[37] Yetiser S., Ince D. (2022). Analysis of persistent geotropic and apogeotropic positional nystagmus of the lateral canal benign paroxysmal positional vertigo. J. Otol..

[38] Legois Q., Molinier C.-E., Nieto P., Marx M. (2024). Repositioning chair treatment procedure for cupulolithiasis: Case report (with video). Eur. Arch. Oto-Rhino-Laryngol..

[39] Dutta K.K., Neupane A.K., Lodha V. (2024). Diagnosis and Management of Trauma Induced Unilateral Horizontal Canal BPPV with Apogeotropic Nystagmus in an Adolescent. Indian J. Otolaryngol. Head Neck Surg..

[40] Martellucci S., Malara P., Castellucci A., Pecci R., Giannoni B., Marcelli V., Scarpa A., Cassandro E., Quaglieri S., Manfrin M.L. (2020). Upright BPPV Protocol: Feasibility of a New Diagnostic Paradigm for Lateral Semicircular Canal Benign Paroxysmal Positional Vertigo Compared to Standard Diagnostic Maneuvers. Front. Neurol..

[41] Maas B.D.P.J., van Leeuwen R.B., Masius-Olthof S., van Benthem P.P.G., Bruintjes T.D. (2021). Treatment Results of Geotropic and Apogeotropic Horizontal Canal Benign Paroxysmal Positional Vertigo in a Tertiary Dizziness Clinic. Front. Neurol..

[42] de Linera-Alperi M.A., Garaycochea O., Calavia D., Terrasa D., Pérez-Fernández N., Manrique-Huarte R. (2022). Apogeotropic Horizontal Canal Benign Paroxysmal Positional Vertigo: Zuma e Maia Maneuver versus Appiani Variant of Gufoni. Audiol. Res..

[43] Maramattom B.V., Sreeram P. (2022). Central Apogeotropic Nystagmus Mimicking A Horizontal Canal “Cupulolithiasis” BPPV. Neurol. India.

[44] Navarro P.P., López S.P., Ayerve C.N.A., Alonso S.M., López J.M.S., Ruiz S.S.C., Sánchez J.C.G., Kaski D., Caletrío Á.B. (2023). Early Diagnosis of Central Disorders Mimicking Horizontal Canal Cupulolithiasis. Brain Sci..

